# Healthy life gains in South Australia 1999-2008: analysis of a local Burden of Disease series

**DOI:** 10.1186/1478-7954-9-13

**Published:** 2011-05-16

**Authors:** David Banham, Tony Woollacott, John Lynch

**Affiliations:** 1 Research and Ethics Policy, SA Health, 11 Hindmarsh Square, Adelaide, SA, 5000, Australia; 2 Division of Health Sciences, University of South Australia, GPO Box 2471, Adelaide, South Australia 5001, Australia

## Abstract

**Background:**

The analysis describes trends in the levels and social distribution of total life expectancy and healthy life expectancy in South Australia from 1999 to 2008.

**Methods:**

South Australian Burden of Disease series for the period 1999-2001 to 2006-2008 and across statistical local areas according to relative socioeconomic disadvantage were analyzed for changes in total life expectancy and healthy life expectancy by sex and area level disadvantage, with further decomposition of healthy life expectancy change by age, cause of death, and illness.

**Results:**

Total life expectancy at birth increased in South Australia for both sexes (2.0 years [2.6%] among males; 1.5 years [1.8%] among females). Healthy life expectancy also increased (1.4 years [2.1%] among males; 1.2 years [1.5%] among females). Total life and healthy life expectancy gains were apparent in all socioeconomic groups, with the largest increases in areas of most and least disadvantage. While the least disadvantaged areas consistently had the best health outcomes, they also experienced the largest increase in the amount of life expectancy lived with disease and injury-related illness.

**Conclusions:**

While overall gains in both total life and healthy life expectancy were apparent in South Australia, gains were greater for total life expectancy. Additionally, the proportion of expected life lived with disease and injury-related illness increased as disadvantage decreased. This expansion of morbidity occurred in both sexes and across all socio-economic groups.

This analysis outlines the continuing improvements to population health outcomes within South Australia. It also highlights the challenge of reducing population morbidity so that gains to healthy life match those of total life expectancy.

## Background

Improving the health of all Australians is an overarching goal of the National Health and Hospitals Network Agreement [1]. The Agreement also outlines commitments to planning activities at the population level and monitoring outcomes across jurisdictions from national to local network areas, by Indigenous and non-Indigenous status, and by socioeconomic disadvantage. This implies the need to track the effect of implemented health reforms and to monitor health status and outcomes at each of these levels [2].

Summary measures of population health help meet this monitoring need. For example, life expectancy is often used in public discussion because of its intuitive appeal, but it is based solely on age-specific mortality and does not address morbidity. Life expectancy measures can now be extended into measures of healthy life expectancy, which account for both length and quality of life [3]. Knowing whether, and to what extent, quality of life is being traded off for quantity of life [4,5] is fundamentally important information for governments, health services, health practitioners, and the public.

Internationally, healthy life expectancy measures are increasingly used as a standard for population health measurement [5,6]. Australian healthy life expectancy estimates have been derived within the national Burden of Disease studies [3,7], which describe morbidity in terms of the amount and severity of disability or illness associated with disease and injury. To enable this, the Australian Burden of Disease studies collated base epidemiological datasets comprising a wide range of disease and injury conditions and their respective parameters (incidence, prevalence, duration), condition sequelae, and disability, or severity, weights. The studies also provided methods for calculating estimates and promoted capacity building for related work and knowledge sharing across jurisdictions. In turn, these factors facilitated South Australia's Strategic Plan inclusion of population targets using healthy life expectancy with routine reporting of state and regional outcomes [8].

This commitment to track healthy life expectancy change over time informs public discussion and planning activities within government. Several cross-sectional analyses of healthy life expectancy have been used to date. For example, cause-deleted analyses of healthy life expectancy [9] help scope potential gains, showing if death and illness from coronary heart disease were averted, healthy life expectancy at birth would increase by around 1.8 years in South Australia. Similarly, intrastate decomposition analyses [10] inform targeted activity. For instance, if cardiovascular disease outcomes in the most socioeconomically disadvantaged areas improved to equal those of least disadvantage, then healthy life expectancy at birth would increase by more than 0.9 years [11] in the most socio-economically disadvantaged areas. To date, analyses of healthy life expectancy changes by age and cause over time have not been used in Australia.

Having developed a time series of healthy life expectancy for South Australia, the current analysis:

• Describes recent trends in total life and healthy life expectancy by sex within South Australia

• Decomposes the contribution of age and cause of death to changes in healthy life expectancy by sex and socio-economic disadvantage

## Methods

The descriptive epidemiology and outcome estimates derived in the Burden of Disease and Injury in Australia: 2003 study [3] provide the base for South Australia's summary population health measurement. Using the South Australian component of those results, a South Australian time series uses annual unit record mortality data and adjustments to morbidity parameters based on yearly changes to sex and age group rates for conditions where relevant administrative data are routinely available. Annually updated, routine data include unit records for cancer registrations, birth defects, communicable diseases and sexually transmitted infections, and those relating to inpatient activity in South Australian hospitals, which are relevant for a range of respiratory and cardiovascular conditions. Additional use is made of South Australian prevalence data for oral health, diabetes, asthma, osteoarthritis, rheumatoid arthritis, gout, and glaucoma taken from cross-sectional population surveys [12,13]. As with the listed administrative unit record sources, these survey data inform the distribution of the respective conditions within South Australia [8].

Initial results for statistical local areas (SLAs), the 127 small geographic areas within South Australia (mean population size of 12348 [SD = 9922] and range from 0 to 35947) were derived using national level synthetic estimates for sex, age, and condition outcomes by quintile of area disadvantage [14] and geographic remoteness. Where local data are available, results were overwritten to distribute the state total to SLAs (and hence area disadvantage quintiles).

The resulting South Australian Burden of Disease and Injury series includes results for the period 1999 to 2008. The outlined consistency in method and approach to annual revisions of age, sex, and condition outcomes enables for valid comparison of results across time. In this particular case, comparison focuses on averaged results for three-year time periods (1999-2001 and 2006-2008).

Abridged period life expectancy tables were constructed from the prevailing age- and sex-specific mortality rates for contiguous three-year periods starting in 1999-2001. Estimates of the age-specific and severity-weighted morbidity prevalence associated with disease and injury were added to the life table using Sullivan's Method [15]. These continuous morbidity measures are represented by the prevalent years lost due to disability (PYLD) [16]. PYLD for each condition and its sequelae comprise the product of each condition's and sequelae's prevalence and severity weighting. The sum for all conditions summarizes total population morbidity for each sex and age group.

Healthy life expectancy results indicate the average number of extra years a person of a particular age and sex might expect to live in full health if current age- and sex- specific mortality and morbidity rates continued for the remainder of their life.

The difference between total life expectancy (LE) and healthy life expectancy (HLE) represents the amount of life expectancy lost to disease and injury-related illness, or expected loss due to illness (ELI). ELI is expressed as a percentage of life expectancy:

Healthy life expectancy change by age was decomposed using a stepwise replacement algorithm [17]. An abridged life table illustrating this approach is available on the South Australian Burden of Disease website [8]. Overall decomposition of change by disease and injury used methods developed by Stephen Begg for Queensland Health [10] within Stata [18] statistical software. The contribution of mortality and morbidity to healthy life expectancy change was partitioned out using stepwise replacement of mortality and morbidity rates.

## Results

### South Australia

**Figure 1:**
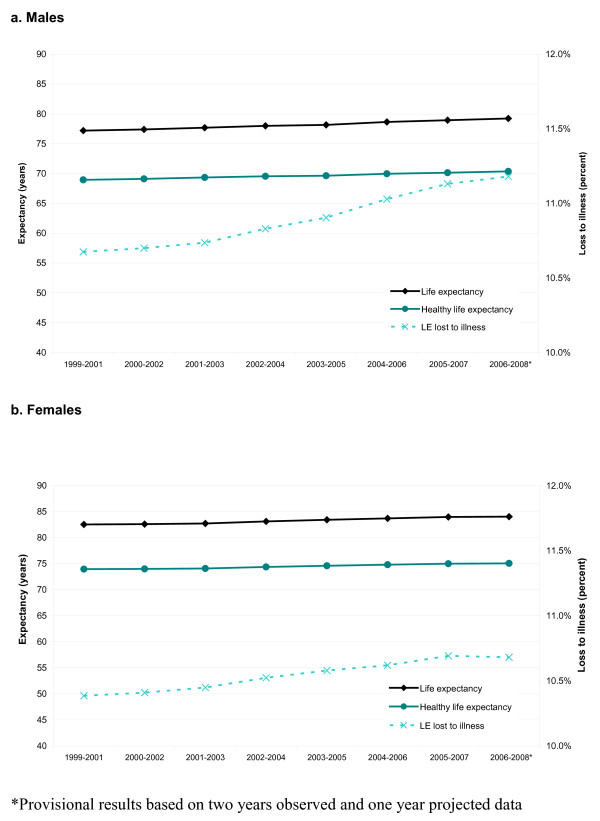
**Life and healthy life expectancy at birth across time by sex in South Australia**.

Figure 1 shows that from 1999-2001 to 2006-2008, life expectancy at birth increased in South Australia by 2.03 years (2.6%) among males and 1.51 years (1.8%) for females. In the same period, healthy life expectancy also increased 1.42 years (2.1%) for males and 1.11 years (1.5%) among females. Given the rate of increase in total life expectancy is greater than that of healthy life expectancy, the percentage of life expectancy lost to severity-weighted illness (ELI) increased over the observed period for both sexes. For males, ELI increased from 10.7% to 11.2% (a relative increase of 4.7%). The corresponding figures among females were 10.4% to 10.7% (a 2.8% relative increase).

In the reported period, improved mortality outcomes added 1.47 years to the change in male healthy life expectancy with approximately 0.05 years (3.4%) of this gain lost to increased morbidity. Improved mortality rates among females added 1.10 years to healthy life expectancy with nominal change (less than 0.01 years, or less than 1.0%) attributed to overall morbidity rates.

### Decomposition by age

#### Males

**Figure 2:**
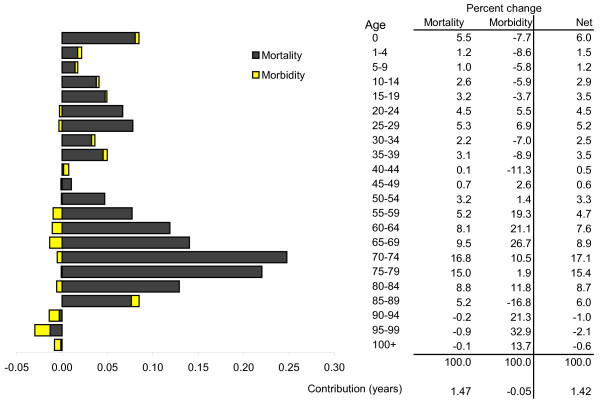
**Contribution of age to changed healthy life expectancy in South Australian males 1999-2001 to 2006-2008**.

Figure 2 shows the age distribution of mortality, morbidity, and overall healthy life expectancy change among males. For example, the 70 to 74 year old year age group added most with 0.24 years (17.1%) of total change. This comprised a quarter of a year from lower mortality rates (16.8% of all mortality change) and a partial offset of < 0.01 year from increased morbidity, which was 10.5% of all morbidity change.

Healthy life gains from mortality improvements were concentrated in ages 60 to 84. Each age group in this range contributed at least 0.1 year toward their collective sum of 0.86 years (58.2% of all gain from mortality). However, this is partially offset by increased morbidity among males aged 55 or more, which held back healthy life expectancy gains by 0.07 years (4.9%). Half of this loss (0.03 years) was concentrated in ages 90 plus. Contrary to this, males up to age 50 contributed 0.02 years (43.9%) to morbidity-based healthy life gain.

Overall, around one-third (32.0%, or 0.45 years) of healthy life expectancy improvement came from ages 0 to 49, one-third (32.5%) was concentrated in the 70 to 79 year age range, and the remaining third across other middle and older age groups.

#### Females

**Figure 3:**
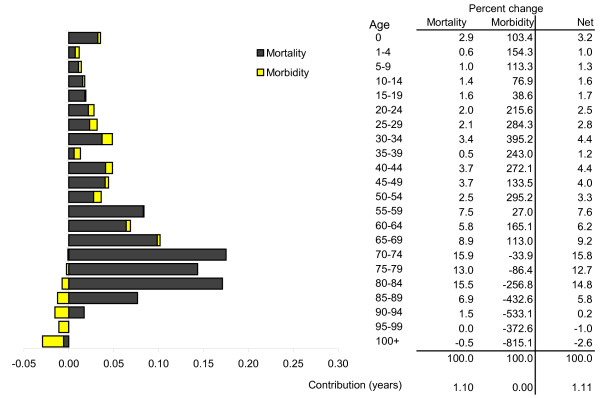
**Contribution of age to changed healthy life expectancy in South Australian females 1999-2001 to 2006-2008**.

The age distribution of mortality, morbidity, and overall change in healthy life expectancy among females is summarized in Figure 3. Again, the 70 to 74 year old group added most to healthy life expectancy with 0.17 years (15.8%) of total change. This was made up of 0.18 years from improved mortality outcomes (15.9% of mortality total) and a partial offset by a very small increase in morbidity.

Healthy life gains due to improved mortality were concentrated in ages 65 to 84. Each age group in this range contributed at least 0.1 year toward their collective sum of 0.59 years (53.3% of all gain from mortality). While, on the whole, female healthy life expectancy change was largely unaffected by morbidity changes, there were notable changes for particular age groupings. Morbidity-related gains up to age 69 amounted to 0.08 years, and an almost equivalent amount (0.07 years) was lost among ages 70 or more.

Overall, more than half (52.5%, or 0.58 years) of healthy life gain came from ages 65 to 84 with one-quarter (25.5%, or 0.28 years) in the 40 to 64 year age range.

### Decomposition by cause

#### Males

**Figure 4:**
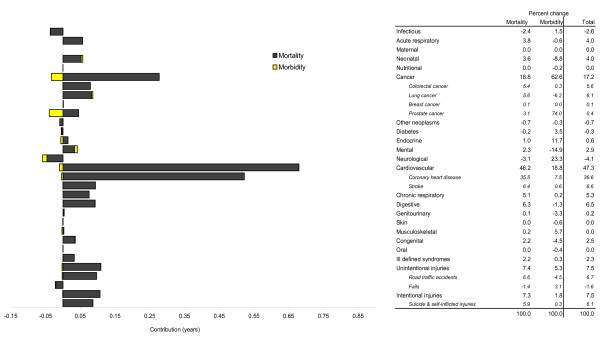
**Contribution of disease and injury categories to changed healthy life expectancy at birth among South Australian males from 1999-2001 to 2006-2008**.

Figure 4 summarizes the contribution of disease and injury categories and selected major conditions to changed healthy life expectancy at birth among South Australian males from 1999-2001 to 2006-2008. For example, 0.68 years (46.2%) of mortality-related gain resulted from reduced cardiovascular disease and coronary heart disease. Improved cancer and injury-related mortality accounted for a further 0.28 years (18.8%) and 0.22 years (14.7%) of healthy life expectancy gain, respectively. There is also evidence of mortality from other causes negatively affecting health expectancy. For example, neurological conditions (including dementia) reduced healthy life change by 0.05 years (3.3%). Increased morbidity detracted 0.05 years (3.8%) from net healthy life gain, and this loss was concentrated among cancers, particularly prostate cancer, which accounted for almost three-quarters (74.0%, or 0.04 years) of the total.

#### Females

**Figure 5:**
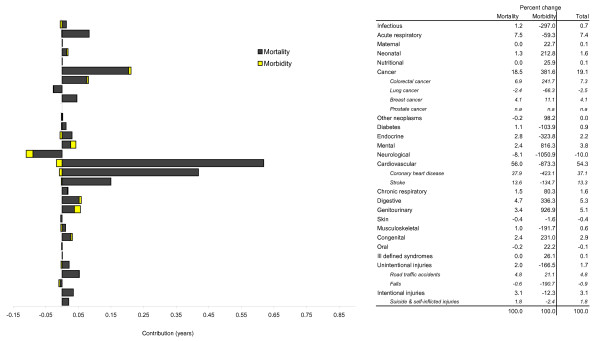
**Contribution of disease and injury categories to changed healthy life expectancy at birth among South Australian females from 1999-2001 to 2006-2008**.

Disease- and injury-related change in healthy life expectancy at birth for South Australian females is summarized in Figure 5. More than half (56.0%, or 0.62 years) of mortality-related improvements to health expectancy resulted from reduced cardiovascular disease, with a further 0.20 years (18.5%) attributed to reduced impact of cancer deaths. There is also evidence of neurological conditions negatively affecting health expectancy, by holding back potential gains by 0.09 years (8.1%). Small increases to morbidity from neurological conditions and cardiovascular disease (0.04 years combined) and similar lowering of morbidity associated with mental health and genitourinary conditions underlie minimal net change in morbidity. Of the additional time gained in the equivalent of full health among females overall, almost three-quarters was attributed to improved outcomes in cardiovascular disease and cancer (54.3% and 19.1% [0.60 and 0.21 years], respectively).

### Decomposition by area disadvantage

Table 1 shows total life expectancy and healthy life expectancy gains are also apparent in each quintile of area level socioeconomic disadvantage. For example, average female life expectancy in the least disadvantaged quintile (Q5) increased by 1.89 years (2.3%) from 1999-2001 to 2006-2008. Female healthy life expectancy in the same quintile also increased, albeit by a smaller amount of 1.27 years (1.7%). Accordingly, the ELI proportion for this group increased by 0.5%, from 9.2% in 1999-2001 to 9.7% in 2006-2008.

**Table 1:** Life and healthy life expectancy at birth across time, sex, and area disadvantage in South Australia

	Life Expectancy (LE)					Healthy Life Expectancy (HLE)					Life Expectancy lost to illness (ELI)				
Males															
3 yearly	Most disadvantage				Least disadvantage	Most disadvantage				Least disadvantage	Most disadvantage				Least disadvantage
average period	**Q1**	**Q2**	**Q3**	**Q4**	**Q5**	**Q1**	**Q2**	**Q3**	**Q4**	**Q5**	**Q1**	**Q2**	**Q3**	**Q4**	**Q5**
1999-2001	74.86	76.53	77.90	78.09	78.93	65.87	68.01	69.45	70.15	71.68	12.0%	11.1%	10.9%	10.2%	9.2%
2000-2002	75.04	76.56	77.90	78.52	79.30	66.08	67.99	69.45	70.42	72.02	11.9%	11.2%	10.8%	10.3%	9.2%
2001-2003	75.46	76.60	78.18	78.61	80.00	66.41	68.04	69.69	70.47	72.57	12.0%	11.2%	10.9%	10.3%	9.3%
2002-2004	75.84	76.75	78.32	79.18	80.23	66.63	68.13	69.73	70.87	72.79	12.2%	11.2%	11.0%	10.5%	9.3%
2003-2005	76.02	76.89	78.51	79.17	80.57	66.72	68.28	69.79	70.84	72.94	12.2%	11.2%	11.1%	10.5%	9.5%
2004-2006	76.61	77.35	79.04	79.62	80.90	67.17	68.58	70.13	71.19	73.10	12.3%	11.3%	11.3%	10.6%	9.6%
2005-2007	76.77	77.70	79.54	79.73	81.19	67.25	68.83	70.51	71.23	73.20	12.4%	11.4%	11.4%	10.7%	9.8%
2006-2008*	77.35	78.10	79.58	79.93	81.47	67.73	69.13	70.60	71.34	73.34	12.4%	11.5%	11.3%	10.7%	10.0%
Change (years)	2.49	1.57	1.67	1.84	2.54	1.86	1.11	1.14	1.19	1.66	0.63	0.46	0.53	0.65	0.88
Change (%)	3.3%	2.0%	2.1%	2.4%	3.2%	2.8%	1.6%	1.6%	1.7%	2.3%	3.5%	3.2%	4.0%	5.7%	8.6%
**Females**															
	**Q1**	**Q2**	**Q3**	**Q4**	**Q5**	**Q1**	**Q2**	**Q3**	**Q4**	**Q5**	**Q1**	**Q2**	**Q3**	**Q4**	**Q5**
1999-2001	81.26	82.01	83.39	83.15	83.21	71.67	73.18	74.54	75.12	75.55	11.8%	10.8%	10.6%	9.7%	9.2%
2000-2002	81.15	82.18	83.44	83.27	83.23	71.61	73.29	74.56	75.19	75.55	11.8%	10.8%	10.6%	9.7%	9.2%
2001-2003	81.18	82.15	83.40	83.48	83.69	71.59	73.24	74.55	75.35	75.90	11.8%	10.8%	10.6%	9.7%	9.3%
2002-2004	81.51	82.50	83.77	83.99	84.06	71.79	73.51	74.86	75.69	76.17	11.9%	10.9%	10.6%	9.9%	9.4%
2003-2005	81.80	82.72	83.92	84.59	84.33	72.04	73.65	75.00	76.14	76.33	11.9%	11.0%	10.6%	10.0%	9.5%
2004-2006	82.04	82.95	84.48	84.71	84.44	72.34	73.80	75.40	76.22	76.38	11.8%	11.0%	10.7%	10.0%	9.6%
2005-2007	82.44	83.22	84.83	84.77	84.75	72.74	73.98	75.58	76.18	76.60	11.8%	11.1%	10.9%	10.1%	9.6%
2006-2008*	82.78	83.43	84.59	84.44	85.10	73.01	74.21	75.44	75.93	76.83	11.8%	11.1%	10.8%	10.1%	9.7%
Change (years)	1.52	1.42	1.20	1.29	1.89	1.34	1.03	0.89	0.80	1.27	0.18	0.40	0.30	0.48	0.62
Change (%)	1.9%	1.7%	1.4%	1.5%	2.3%	1.9%	1.4%	1.2%	1.1%	1.7%	0.0%	2.7%	2.0%	4.4%	5.7%

*Provisional results based on two year observed and one year projected data

The distribution of gains also varied by sex and area disadvantage quintile. Among males, the largest life expectancy increases were in quintiles of most and least disadvantaged, each with around 2.5 years. Similarly, healthy life expectancy increased differentially, whereby the most disadvantaged areas (Q1) gained 1.86 years and those of least disadvantage (Q5) gained 1.66 years. Further, ELI increased in all quintiles with relative loss in the least disadvantaged areas doubling that of the most disadvantaged areas.

As with males, female healthy life expectancy increased most (around 1.3 years) in areas of most and least disadvantage. Underlying this were differences in life expectancy gains, with increases of 1.52 years (1.9%) in the most disadvantaged areas and 1.89 years (2.3%) in the least disadvantaged areas. The proportion of ELI did not change for the most disadvantaged but increased by 0.5% (0.62 years) to 9.7% (8.27 years) in the least disadvantaged.

### Decomposition by age and area disadvantage

**Figure 6:**
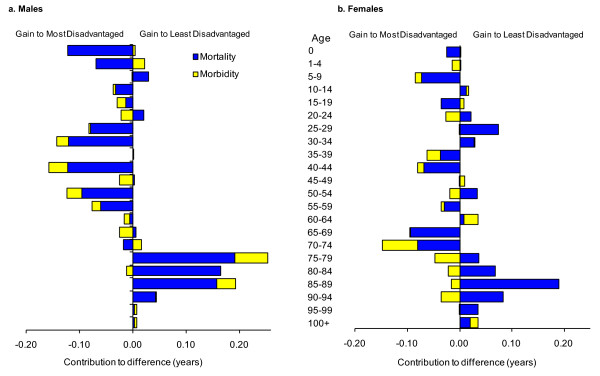
**Difference in age contribution to changed Healthy Life Expectancy in areas of least and most disadvantage from 1999-2001 to 2006-2008**.

Figure 6 compares healthy life expectancy outcomes in areas of most (Q1) and least (Q5) disadvantage by sex. Bars falling to the left of the vertical axis indicate relative gains in the most versus least disadvantaged areas, while those to the right indicate relative gains were greater amongst the least versus most disadvantaged areas.

Up to the age of 64, males in the most disadvantaged areas had larger healthy life expectancy gains than those in the least disadvantaged areas. Within this age range, disadvantaged males' additional healthy life expectancy of 0.83 years was made up of 0.67 years (80.7%) mortality gain and 0.16 years (19.3%) of morbidity-related gain. Conversely, in ages 75 and over, the least disadvantaged males had 0.66 years more healthy life expectancy gain than their more disadvantaged counterparts. This comprised 0.56 years (84.8%) from mortality and 0.10 years (15.2%) from morbidity.

The pattern among females was similar for mortality outcomes. Ages up to 74 years in the most disadvantaged areas experienced more positive change compared to the least disadvantaged areas (a total of 0.26 years). However, in ages 75 and over, the least disadvantaged females gained 0.43 years more than their less advantaged counterparts.

Morbidity outcomes differed in that the most disadvantaged females experienced comparatively more positive change compared to those in least disadvantaged areas over most age groups.

### Decomposition by cause and area disadvantage

**Figure 7:**
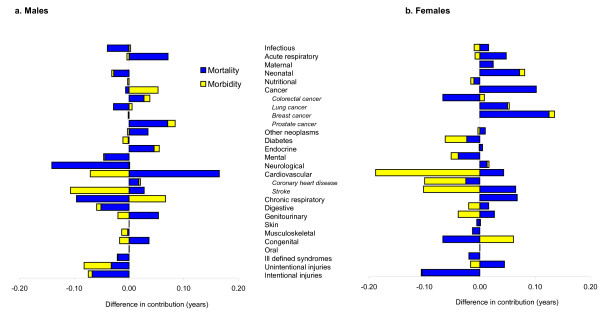
**Difference in disease and injury category contribution to changed Healthy Life Expectancy in areas of least and most disadvantage from 1999-2001 to 2006-2008**.

Figure 7 also compares healthy life expectancy outcomes observed in areas of most and least disadvantage but collapses results by disease and injury categories. Bars to the left of the vertical axis again indicate relative gains in the most versus least disadvantaged areas and vice versa.

Among males, the largest area of difference between results for most versus least disadvantage was cardiovascular-related mortality. In this particular case, both quintiles gained healthy life expectancy, but the least disadvantaged males gained 0.17 years more than their contemporaries in areas of the most disadvantage. Of the gains in the most disadvantaged areas, 0.73 years (39.5%) was due to cardiovascular mortality, compared to 0.89 years (51.9%) in the least disadvantaged areas. Mortality-related change due to neurological conditions was comparatively better in disadvantaged areas, but this was due to increased mortality from neurological conditions among the least disadvantaged. Improved injury outcomes also led to comparatively larger healthy life expectancy gains for males in the most disadvantaged areas.

Among females, gains from intentional injury were higher by 0.11 years in most versus least disadvantaged areas. However, the most striking difference between results for most and least disadvantage is in cardiovascular-related morbidity (0.19 years). In this case, a morbidity decrease of 0.10 years in areas of most disadvantage contributed 7.5% of healthy life expectancy gain, while an 0.09 year increase in morbidity in the least disadvantaged areas retarded healthy life expectancy gain by a further 7.1%.

## Discussion

Both life expectancy and healthy life expectancy in South Australia showed steady improvement from 1999 to 2008. Improvements to healthy life expectancy were particularly influenced by reduced death rates in ages 60 to 84 years for males. While the degree of mortality improvement was higher among males than females, females experienced improved mortality outcomes across a narrower age range from 65 to 84. Lower mortality rates from cardiovascular disease and cancers contributed substantially to improve outcomes in both sexes. However, with deaths increasingly deferred to older ages, neurological conditions, including dementia, became more prominent. Changes due to morbidity were generally very small, with healthy life expectancy losses confined to males above age 55 and females above age 75. In the examples of cardiovascular disease and cancers, results point to considerable gains in healthy life expectancy, with an underlying transition away from death by cardiovascular disease and cancers toward life as a survivor of one of these illnesses.

All socioeconomic groups showed improvements in life and healthy life expectancy, with the largest gains at the extremes of the socioeconomic spectrum. The estimates also show that the absolute gap between total life and healthy life expectancy improvements remains highest in areas of most disadvantage. There was no clear trend in the gap between outcomes for most and least disadvantaged areas widening or closing. However, taken as a whole, the gap between each quintile's total life expectancy and healthy life expectancy tended to increase as area disadvantage decreased. In other words, more advantaged areas had larger increases in the gaps between length of life (total life expectancy) and quality of life (healthy life expectancy), but this trend was small. Comparing the extreme quintiles for females, this was influenced by increased morbidity associated with chronic disease among older groups in areas of least disadvantage. Similar comparison of male outcomes suggests increased morbidity in older ages, regardless of quintile. However, the results also point to increased morbidity among middle-aged males in areas of least disadvantage. This was associated with chronic diseases such as stroke, diabetes, and diagnosed prostate cancer.

The most disadvantaged quintile's comparatively large increases in total life and healthy life expectancy are unexpected and deserve closer scrutiny. First, revised life expectancy figures for this group for later periods will be slightly reduced when the next deaths data become available and the series' annual update completed. This is because death records are processed by year of death rather than year of registration, and a number of death records in latter years are not available until the completion of coroner's inquiries. Previous analysis indicates these extra deaths will be concentrated in more disadvantaged areas [11]. Numerator adjustment is only a partial explanation, though. It is possible that population profiles change at different rates among quintiles. Such change may be influenced by the resettlement of healthy, working age migrants within areas of lower socioeconomic position, which would reduce mortality rates per capita in working ages. This notion is consistent with comparatively larger healthy life expectancy gains being observed among younger, working age people in areas of most disadvantage. This uncertainty in interpretation highlights the need for further investigation into intrastate population movement and the implications for health planning and policy, particularly within localized health networks.

Consistent with previously published research [4], South Australian estimates show not all life years gained are "full of health." With life expectancy increasing at a higher rate than healthy life expectancy, the amount of life expected to be lived with disease and injury-related illness increased over time and suggests a relative expansion of morbidity in the South Australian population, particularly in older age groups. The observed increase in the amount of life lived but spent with illness was consistent for males and females and within each socioeconomic quintile, with the exception of females in areas of most disadvantage. In that particular case, there was no apparent change in the proportion of life lost to illness. This suggests that, for the most disadvantaged quintile, a morbidity gain of 0.20 healthy life years (the largest observed positive result) was sufficient to equalize the relative trajectories of life and healthy life expectancies. It is possible for an absolute reduction in time lost to morbidity, or absolute compression of morbidity, to occur contemporaneously with a relative expansion of morbidity. This points to a further line of analysis focused on estimating the threshold improvement to morbidity required to equalize the rate of change of healthy life expectancy in light of projected life expectancy change.

There are limitations to these data that should be acknowledged. First, it is not possible to examine the precision of time trends, as there is currently no way to gauge confidence intervals around the morbidity point estimates in Burden of Disease studies. Monte Carlo simulation and sensitivity analyses may inform future solutions to this issue. Another line of analysis is to compare changes to Burden of Disease PYLD point estimates with those observed in health utility measures within community samples in South Australia's Health Omnibus Surveys across a similar time frame. The latter survey results provide uncertainty intervals around utility point estimates and will inform on the degree to which morbidity change is random or not. Second, no new morbidity-related data were introduced to important areas such as mental health. This is a serious omission, but the challenges of developing and maintaining condition-specific models are difficult for a single jurisdiction. A remedy is to develop a systematic, national work program devoted to routine development and production of summary population health measures and exploring their potential for evaluating outcomes from health-related activities. Further, considerable use was made of hospital inpatient records. By nature, these reflect hospital separations, and the age-specific rate changes noted for a range of conditions may reflect service volume change rather than incident episodes for patients. Statistical data linkage of administrative records has the potential to greatly enhance our ability to denote changing incidence on a per capita basis [4].

## Conclusions

This analysis highlights the health and social system challenges of attempting to ensure that added years of life are healthy and distributed with social equity. Most life expectancy gains tend to be partially offset by illness-related loss, which, in turn, produces demand on resources. These results point to the need for national policy debates to look beyond simple life expectancy trends toward the health-related quality of extra years of life and how they are distributed across socioeconomic groups. Australia has at once a rapidly aging and a rapidly growing population, and this debate has significant implications for future workforce participation and health care demands.

## Competing interests

The authors declare that they have no competing interests.

## Authors' contributions

DB maintains the South Australian Burden of Disease series, conceived of the study, performed all data analysis, and drafted and revised the manuscript. TW participated in the study design and helped draft and revise the manuscript. JL contributed to the study design and aspects of decomposition methodology and helped draft and revise the manuscript. All authors read and approved the final manuscript.
